# Peroxidase as the Major Protein Constituent in Areca Nut and Identification of Its Natural Substrates

**DOI:** 10.1155/2013/412851

**Published:** 2013-10-24

**Authors:** Yu-Ching Liu, Chao-Jung Chen, Miau-Rong Lee, Mi Li, Wen-Tsong Hsieh, Jing-Gung Chung, Heng-Chien Ho

**Affiliations:** ^1^Department of Medical Research, China Medical University Hospital, Taichung 40402, Taiwan; ^2^Department of Veterinary Medicine, National Chung Hsing University, Taichung 40227, Taiwan; ^3^Graduate Institute of Integrated Medicine, China Medical University, Taichung 40402, Taiwan; ^4^Proteomics Research Core Lab, China Medical University, Taichung 40402, Taiwan; ^5^Department of Biochemistry, China Medical University, Taichung 40402, Taiwan; ^6^Wholesaler Enterprise Company, Taipei 23941, Taiwan; ^7^Department of Pharmacology, China Medical University, Taichung 40402, Taiwan; ^8^Department of Biological Science and Technology, China Medical University, Taichung 40402, Taiwan; ^9^Department of Biotechnology, Asia University, Taichung 41354, Taiwan

## Abstract

Numerous reports illustrate the diverse effects of chewing the areca nut, most of which are harmful and have been shown to be associated with oral cancer. Nearly all of the studies are focused on the extract and/or low molecular weight ingredients in the areca nut. The purpose of this report is to identify the major protein component in the areca nut. After ammonium sulfate fractionation, the concentrated areca nut extract is subjected to DEAE-cellulose chromatography. A colored protein is eluted at low NaCl concentration and the apparently homogeneous eluent represents the major protein component compared to the areca nut extract. The colored protein shares partial sequence identity with the royal palm tree peroxidase and its peroxidase activity is confirmed using an established assay. In the study, the natural substrates of areca nut peroxidase are identified as catechin, epicatechin, and procyanidin B1. The two former substrates are similarly oxidized to form a 576 Da product with concomitant removal of four hydrogen atoms. Interestingly, oxidation of procyanidin B1 occurs only in the presence of catechin or epicatechin and an additional product with an 864 Da molecular mass. In addition, procyanidin B1 is identified as a peroxidase substrate for the first time.

## 1. Introduction

According to the Taiwan Cancer Registry annual report (Department of Health, Executive Yuan, Taiwan) in 2009, the annual incidence of oral malignancy was 6,480 cases with a median age of 53 years, an increase of 699 cases from 2008. Oral cancer had become the fifth leading cause of tumors in Taiwan, preceded by malignancies of the colon, liver, lung, and breast. More than 90% of the new incidences (5927 cases) in 2009 were in males, who engage in chewing the areca nut. For males in Taiwan, this malignancy represented the fourth primary cause of death and occurred at an average age of 55 years. Epidemiological studies and statistical analyses clearly demonstrated that the development of oral cancer in Taiwan was highly associated with chewing the areca nut, smoking, and wine consumption [1]. Chewing the areca nut wrapped in the Piper betle leaf, which is coated with slaked lime, is popular in Taiwan, especially in the southeastern region. It has been estimated that there were at least two million out of 23 million residents in Taiwan who were areca nut chewers. Approximately 90% of oral cancer patients in Taiwan have participated in this practice.

The areca nut is the seed of the palm plant *Areca catechu L.* and is rich in alkaloids, including arecoline, arecaidine, guvacine, and guvacoline. Sundqvist et al. demonstrated that the aqueous extract and the above four alkaloids from the areca nut are highly cytotoxic and genotoxic to human cultured cells and have the potential to induce tumors [2]. Additionally, several reports have shown the association of chewing areca nuts with the development of various disorders, such as cardiovascular disease [3], metabolic syndrome [4], and hypertension [5]. In spite of these various harmful properties, the areca nut is still used in traditional Chinese medicine and in India. It was found to be a highly effective treatment for human taeniasis [6] and increased memory and learning in a rat model [7].

Apart from the numerous studies on small-molecule ingredients in the areca nut, little is known about the protein components, which is the focus of this report. The major protein component in the areca nut was purified as an apparently homogeneous product and found to be a peroxidase, whose activity was subsequently revealed. The natural substrates for the areca nut (AN) peroxidase were also demonstrated in this study.

## 2. Materials and Methods

### 2.1. Reagents

Areca nut was obtained from a local supplier in northern Taiwan. DEAE- (diethylaminoethyl-) cellulose was purchased from Whatman, Inc. (Clifton, NJ). Prestained protein marker was obtained from Fermentas (St. Leon-Rot, Germany). Guaiacol, (+)-catechin, and (−)-epicatechin were obtained from Sigma-Aldrich Corp. (St. Louis, MO). PNGase F was purchased from New England BioLabs (Ipswich, UK). Procyanidins B1 and B2 were obtained from Extrasynthese (Genay, France), and sinapinic acid (SA) and *α*-cyano-4-hydroxycinnamic acid (CHCA) were purchased from Bruker Daltonics, Inc. (Bremen, Germany).

### 2.2. Single-Column Purification of the Major Protein Component in the Areca Nut

The following protocol was performed at room temperature. Areca nut (330 g) was homogenized with 1 liter of buffer A (20 mM Tris/HCl, pH 7.0). The homogenate was filtered and centrifuged at 13,000 rpm for 20 min at 4°C, and the clear supernatant was filtered again. Proteins in the areca nut (AN) extract were fractionated with 80% ammonium sulfate and centrifuged. The protein precipitate was redissolved in buffer A and dialyzed against buffer A overnight. The dialysis step was repeated once, and the dialyzed sample was loaded onto a DEAE-cellulose column preequilibrated with buffer A. After loading, the column was washed with 100 mL of buffer A, and the proteins in the column were eluted with a linear gradient of 1 M NaCl buffer A. A brown-colored protein was eluted at a low concentration of NaCl.

### 2.3. Protein Identification by MALDI-TOF-TOF MS

The protein band on the SDS-PAGE gel was excised, followed by in-gel digestion and peptide extraction according to the protocol described previously [8]. MS spectra were acquired with MALDI-TOF-TOF MS (Ultraflex III TOF/TOF, Bruker Daltonics, Inc.) equipped with a smartbeam laser. For digested peptide analysis, a CHCA matrix (1 mg/mL in 50% acetonitrile containing 0.1% TFA) was mixed with an equal volume of peptide sample on the MALDI sample plate and then air-dried. The spectra were recorded in reflector mode using an acceleration voltage of +20 kV and a 20 ns delay time. Intense MS peaks were selected for MS/MS analysis and peptide mass fingerprinting (PMF). The precursor mass window was set at ±0.45% of the precursor mass. The peak list from the MS spectra was processed by FlexAnalysis v.3.0 software (Bruker Daltonics, Inc.). The extracted peak list was compared against the NCBInr database (release 20101220) using MASCOT v.2.2.04 (Matrix Science, UK). Search parameters were selected as follows: taxonomy-Viridiplantae (green plants); enzyme-trypsin; fixed modification-carbamidomethyl (C); variable modification-oxidation (M, H, W); peptide mass tolerance ± 100 ppm; MS/MS tolerance ± 0.5 Da; and significant threshold *P* < 0.05.

The molecular mass of the purified protein was determined in linear mode. Equal volumes (0.5 **μ**L) of protein sample and SA matrix (saturated in 30% acetonitrile and 0.1% TFA) were mixed on a ground MALDI plate (Bruker Daltonics, Inc.). Protein mass calibration was performed using a protein mixture kit II consisting of protein A [M+2H]^2+^, trypsinogen [M+2H]^+^, and protein A [M+2H]^+^ (Bruker Daltonics, Inc.).

### 2.4. Activity Assay and Zymogram of AN Peroxidase

Activity of AN peroxidase was assayed by measuring the absorbance at 470 nm in 20 mM guaiacol and 5 mM H_2_O_2_ in 0.1 M citrate-phosphate buffer (pH 5.5). For peroxidase zymography, a native gel was used to analyze the purified protein without heating and addition of a reducing agent. After electrophoresis, the gel was rinsed with water and immersed in 20 mM guaiacol and 5 mM H_2_O_2_ in water. A brown color appeared shortly after the addition and indicated the presence of peroxidase.

### 2.5. Digestion of Glycosylated AN Peroxidase with PNGase F

The purified peroxidase (13 *μ*L) was mixed with 2 *μ*L of denaturation buffer (10X) and heated in boiling water for 10 min. The following components were then added to the mixture: 2 **μ**L of G7 reaction buffer (10X), 2 *μ*L of NP-40 (10%), and 1 *μ*L of PNGase F (500 U/*μ*L). The mixture was incubated at 37°C overnight or longer and analyzed by SDS-PAGE. The bands of interest were excised and analyzed by MALDI-TOF-TOF MS as mentioned above.

### 2.6. Detection of Peroxidase Substrate(s) in Areca Nut Extract by LC-MS/MS

Wavelength scanning was initially employed to determine whether peroxidase substrate(s) existed in areca nut extract. In a 500 *μ*L reaction vessel, 50 *μ*L of areca nut extract was mixed with 5 *μ*L of H_2_O, H_2_O_2 _(0.5 M), or guaiacol (2 M), in 0.1 M citrate-phosphate buffer (pH 5.5). The absorbance of the whole mixture was then measured at 380~600 nm.

An Atlantis T3 C_18_ column (5 *µ*m, 2.1 × 150 mm) (Waters Corp., Milford, MA) was used to analyze the above areca nut extract using a linear gradient at a flow rate of 0.25 mL/min. The mobile phases consisted of ddH_2_O (solvent A) and acetonitrile (solvent B), both of which contained 0.1% formic acid (v/v). HPLC separation was performed on a Dionex Ultimate 3000 HPLC system (Thermo Fisher Scientific, Waltham, MA) equipped with a pump (DGP 3600M) and an autosampler (WPS-3000T). An ion trap mass spectrometer with an ESI source (HCTultra PTM Discovery System, Bruker Daltonics, Inc.) was used for LC-MS full scan measurements and LC-MS/MS experiments. The ESI source was operated in positive ion mode with nitrogen as a nebulizing (50 psi) and drying gas (10 L/min, 350°C). MS and MS/MS were done in ultrascan mode in the mass ranges *m/z* 100–800 and *m/z* 50–1000, respectively.

### 2.7. Oxidation of Catechin, Epicatechin, and Procyanidin B1 by AN Peroxidase

The following oxidation reaction was performed in 0.1 M citrate-phosphate buffer (pH 5.5) at room temperature. In a 200 *μ*L reaction vessel, final concentrations of 2 mM H_2_O_2_ and 0.5 mM catechin or epicatechin were mixed with AN peroxidase for wavelength and time scanning and HPLC analysis monitored at 280 or 475 nm. A LiChroCART column (4 × 250 mm) (Merck & Co., Inc., Whitehouse Station, NJ) was used for separation with a linear gradient at a flow rate of 1 mL/min. The above mobile phases (ddH_2_O and acetonitrile) were used; however, no formic acid was added. For oxidation analysis of the third substrate, 0.1 mM procyanidin B1 was used in the presence or absence of 0.2 mM catechin or epicatechin.

## 3. Results

### 3.1. Identification of Peroxidase as the Major Protein Component in the Areca Nut

**Figure 1:**
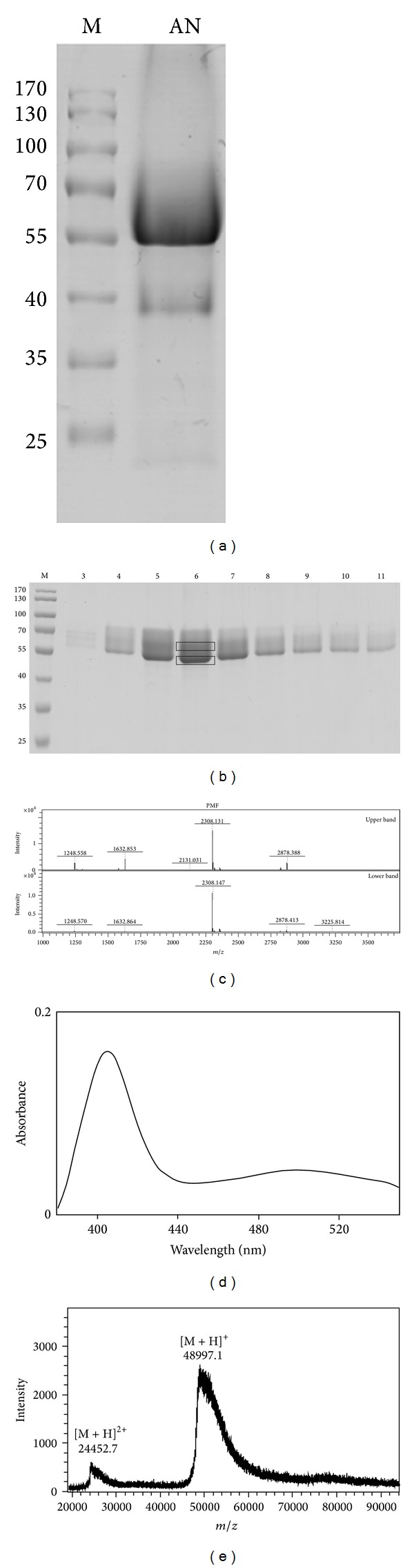
Purification of the major protein component in the areca nut. (a) SDS-PAGE analysis of the concentrated extract. M, prestained protein marker with the molecular weights indicated on the left. AN, areca nut extract. (b) SDS-PAGE analysis of the collected brown-colored fractions from DEAE-cellulose chromatography. The collected fraction number is indicated at the top. The upper and lower bands were bracketed for identification purposes. (c) PMF for the upper and lower bands. The molecular mass of each tryptic peptide is indicated at the top. (d) Wavelength scanning of the purified brown-colored protein. (e) Determination of the molecular mass for the purified protein by MALDI-TOF.

As illustrated in Figure 1(a), SDS-PAGE analysis revealed a major protein product with a molecular mass of ~55 kD in areca nut extract. A brown-colored protein was eluted early at a low concentration of NaCl when performing DEAE-cellulose chromatography. The colored eluent consisted of the major protein product characterized by severe smearing in the SDS-PAGE gel (Figure 1(b)). The homogeneity of the purified protein was further confirmed using PMF to analyze the upper and lower bands in the gel (Figure 1(b)). The same patterns were obtained for both bands (Figure 1(c)). The colored protein exhibited a maximum absorbance near 405 nm (Figure 1(d)), and MALDI-TOF analysis also displayed a smearing pattern with a 49 kD molecular mass (Figure 1(e)).

**Figure 2:**
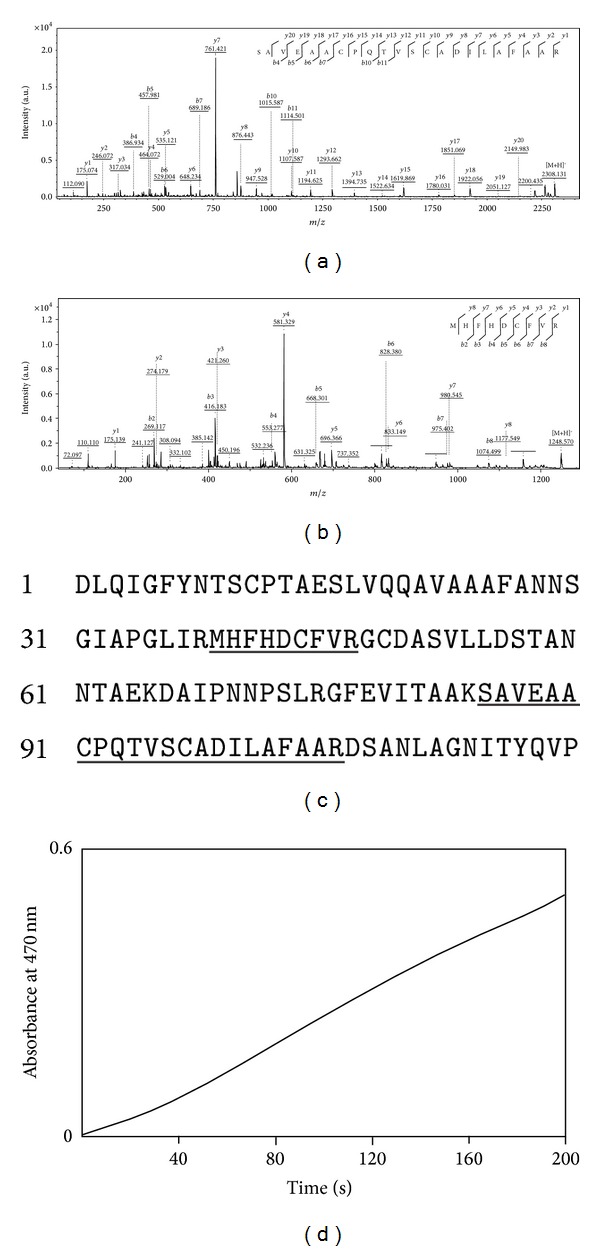
Identification of peroxidase as the major protein component. (a) and (b) MS/MS analysis of two tryptic peptides with the sequences at the top of each panel. (c) Sequence alignment of two tryptic peptides with the N-terminal 120 residues of RPT peroxidase. The sequence identity is underlined, and the numbers on the left indicate the residue numbering of RPT peroxidase. (d) Spectrophotometric assay of peroxidase activity for the purified protein.

MALDI-TOF/TOF mass spectrometric analysis led to the identification of two tryptic peptides with molecular masses of 2308.131 and 1248.57 Da (Figure 1(c)). Sequences of both tryptic peptides (Figures 2(a) and 2(b)) were found to be identical with the amino terminus region of royal palm tree (RPT) peroxidase, as shown by the underlining in Figure 2(c). Peroxidase activity of the purified protein was further illustrated by measuring an absorbance increase at 470 nm using guaiacol as the substrate in the presence of hydrogen peroxide (Figure 2(d)).

### 3.2. Dimerization and Glycosylation of AN Peroxidase

**Figure 3:**
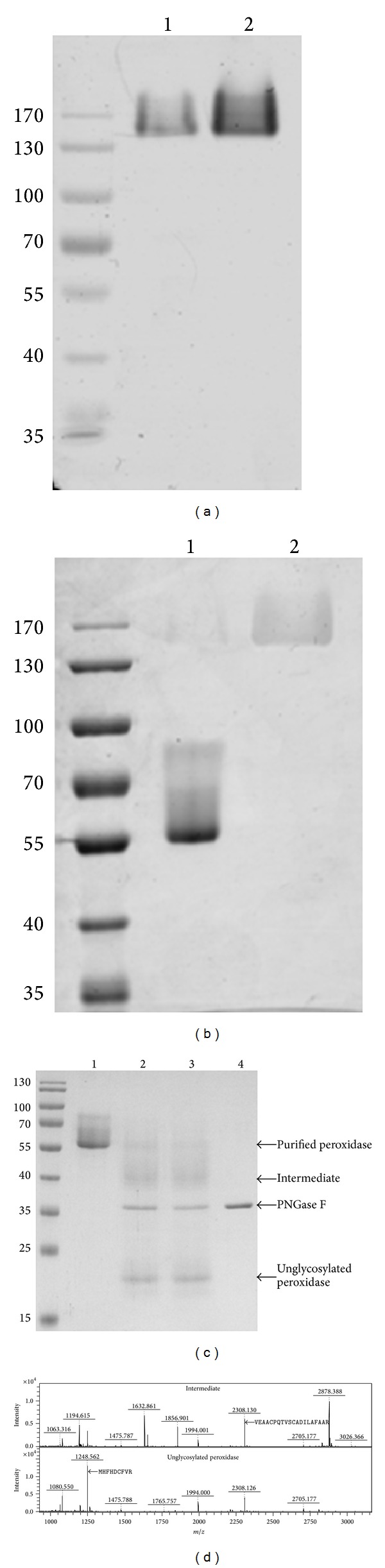
Dimerization and glycosylation of the intact AN peroxidase. (a) Zymogram assay and (b) Coomassie staining. The enzyme was desalted with Tris/HCl buffer (20 mM, pH 7) (1) and HEPES buffer (20 mM, pH 7.9) (2). (c) Treatment of denatured AN peroxidase with PNGase F for 24 (2) and 48 h (3). The digested products, accompanied by the untreated control (1) and PNGase F itself (4), are indicated by arrowheads at the right. (d) PMF for the partially glycosylated intermediate (upper panel) and unglycosylated form (lower panel). Two tryptic peptides subjected to analysis are indicated by arrowheads with the sequences shown in each panel.

When performing peroxidase zymography, unexpectedly slow migration of active peroxidase was observed (Figure 3(a)), but it showed much higher activity at a higher pH (2 in Figure 3(a)). Compared to the Coomassie staining pattern, it clearly showed that lower activity at neutral pH was the result of nearly complete dissociation of the peroxidase subunits into inactive monomers (1 in Figure 3(b)), whereas the interaction between subunits was stable at higher pH (2 in Figure 3(b)). Therefore, the two identical subunits were noncovalently associated to form an intact AN peroxidase with full activity.

AN peroxidase was structurally similar to RPT peroxidase, which has nine N-glycosylation sites [9]. The smearing pattern in the gel of AN peroxidase was most likely the consequence of high glycosylation. To confirm this hypothesis, AN peroxidase was digested to form products with partial or complete removal of N-linked oligosaccharides through 24 and 48 h treatments with PNGase F (2 and 3 in Figure 3(c)). Two separate tryptic peptides in the intermediate and unglycosylated products were identified (Figure 3(d)) with the same sequences as those in Figures 2(a) and 2(b). Clearly, different extents of AN peroxidase glycosylation resulted in a broad spectrum of molecular masses, especially because the glycan moiety was estimated to be more than half of the glycoprotein.

### 3.3. Catechin, Epicatechin, and Procyanidin B1 Are Areca Nut Peroxidase Substrates

**Figure 4:**
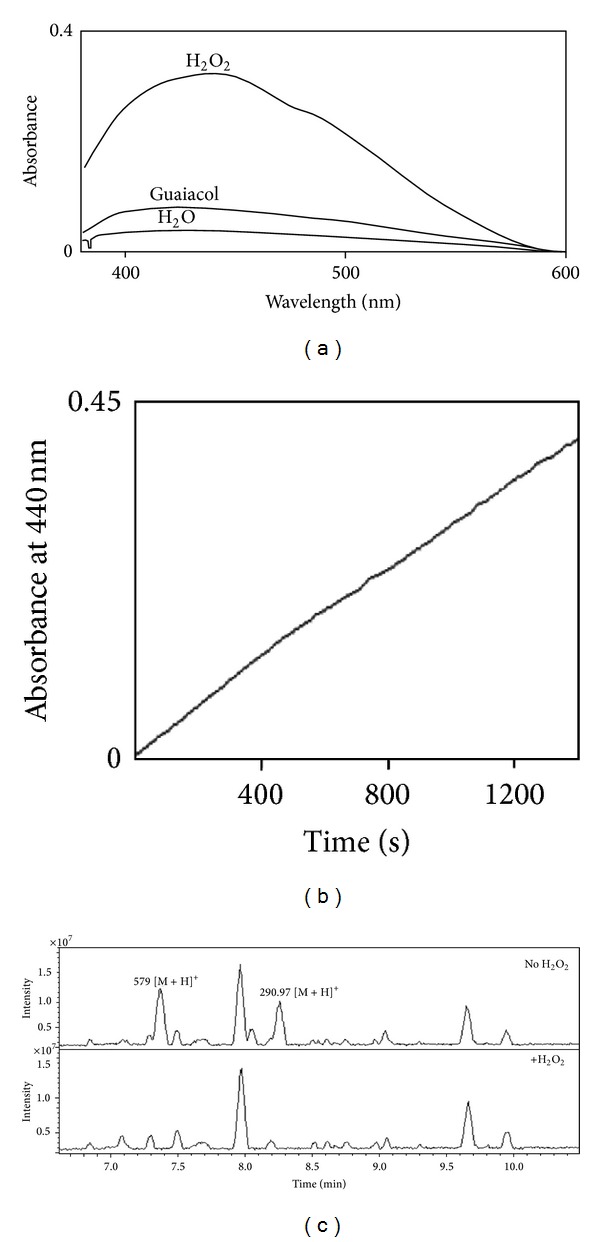
Detection of the natural substrate(s) in the areca nut. (a) Wavelength scanning between 380 and 600 nm after mixing the AN extract with hydrogen peroxide, guaiacol, or water. (b) Measurement of absorbance increase at 440 nm for the AN extract in the presence of hydrogen peroxide. (c) LC/MS analysis of the AN extract in the presence of water (upper panel) or hydrogen peroxide (lower panel).

When hydrogen peroxide was added to the colorless extract of the areca nut, a slow color change from yellow to orange was observed, whereas addition of guaiacol or water had no such effect. Wavelength scanning indicated a maximum absorbance for the mixture at 440 nm (Figure 4(a)), and the absorbance increases at the same wavelength were similar to those observed in the time scanning (Figure 4(b)). Comparison of the LC chromatograms clearly indicated that the differences in the two components were completely removed in the presence of hydrogen peroxide (lower panel in Figure 4(c)). These two candidate substrates eluted at 7.4 and 8.3 min and showed molecular masses of 578 and 290 Da, respectively (upper panel in Figure 4(c)).

**Figure 5:**
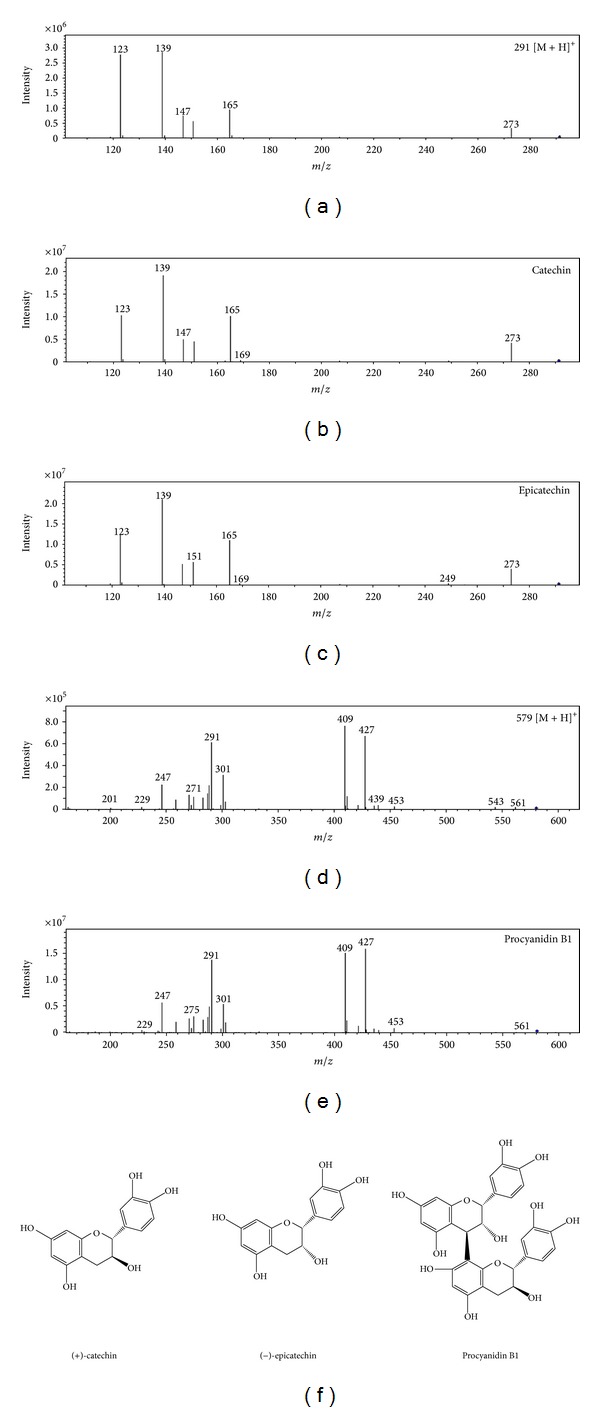
Identification of the natural substrates in the areca nut. (a), (b), and (c) Comparison of the MS/MS spectrum of the 290 Da substrate with those of catechin and epicatechin. (d) and (e) Similarity of the MS/MS spectra between the 578 Da substrate and procyanidin B1. (f) The structures of catechin, epicatechin, and procyanidin B1.

Furthermore, the MS/MS spectrum of the substrate with a 290 Da molecular mass (Figure 5(a)) showed it to be catechin and epicatechin, both of which displayed indistinguishable spectra (Figures 5(b) and 5(c)). On the other hand, fragment ions in the MS/MS spectrum of the substrate with a 578 Da molecular mass (Figure 5(d)) were very similar to those of procyanidin B1 (Figure 5(e)). These three components were recognized as the natural substrates for AN peroxidase. The structures of catechin, epicatechin, and procyanidin B1 were shown in Figure 5(f).

### 3.4. Oxidation of Catechin and Epicatechin by AN Peroxidase

**Figure 6:**
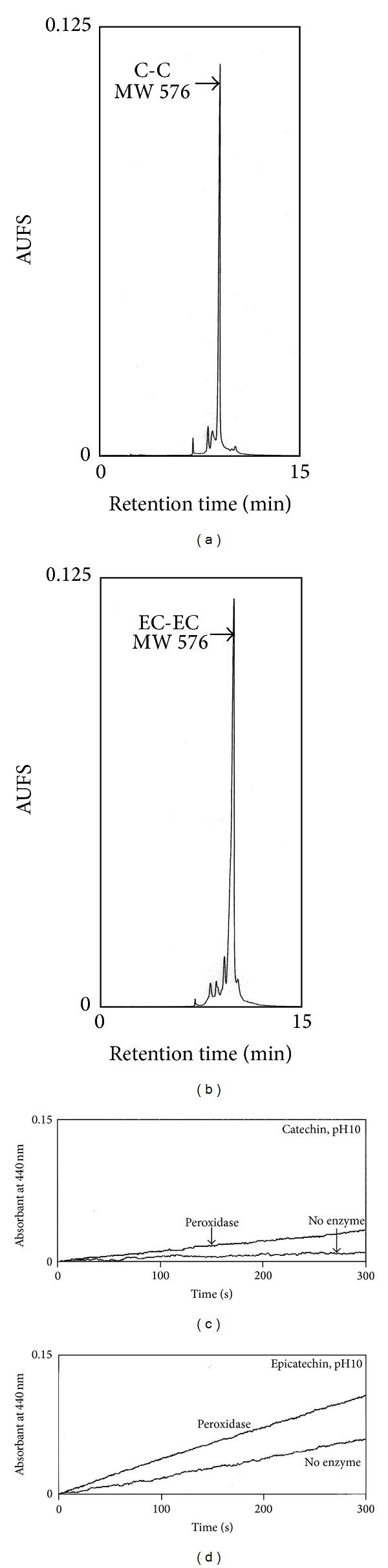
Oxidation of catechin and epicatechin. (a) and (b) HPLC analysis of AN peroxidase-mediated oxidation of catechin (a) and epicatechin (b). The dimerized products with the same molecular mass are identified as C-C and EC-EC. (c) and (d) Spectrophotometric activity assay of AN peroxidase at higher pH using catechin (c) and epicatechin (d) as substrates. The spontaneous oxidation of both substrates in the absence of enzyme is also included.

When performing the oxidation of catechin and epicatechin with hydrogen peroxide, a color change was immediately observed after AN peroxidase addition. The spectra of wavelength scanning for both reactions were similar to that observed for the areca nut extract (Figure 4(a)). HPLC examination of the oxidation reactions revealed that catechin and epicatechin were oxidized to a major product with a distinct retention time (Figures 6(a) and 6(b)). The major oxidized products, with the same molecular mass of 576 Da for both substrates, were undoubtedly the consequence of condensation of two catechin or epicatechin molecules with the concomitant removal of four hydrogen atoms. While chewing the areca nut along with the Piper betle leaf in slaked lime in the oral cavity, the pH value of the collected oral extracts usually fell between 8 and 9, in our experience. AN peroxidase still retained the ability to oxidize catechin and epicatechin at pH 10, although to a lesser extent than at neutral pH (Figures 6(c) and 6(d)). Under these conditions, the substrates were spontaneously oxidized, and AN peroxidase accelerated the oxidation process.

### 3.5. Occurrence of Procyanidin B1 Oxidation in the Presence of Catechin or Epicatechin

**Figure 7:**
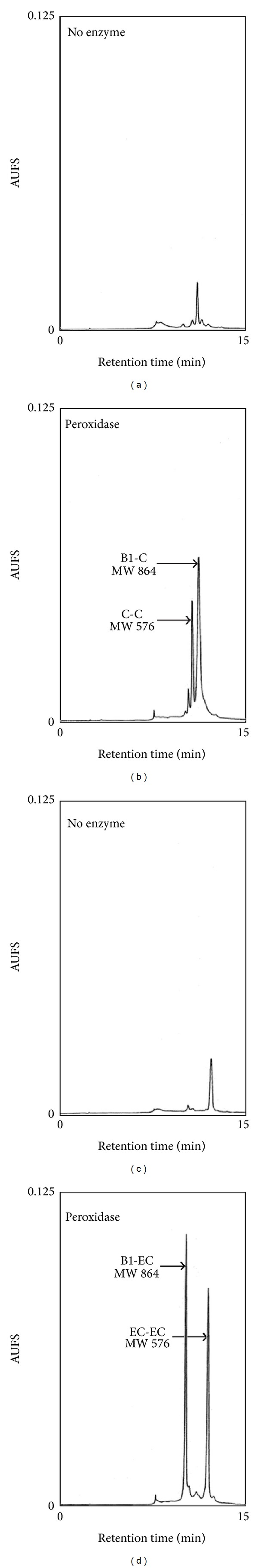
Procyanidin B1 oxidation in the presence of catechin and epicatechin. HPLC chromatograms were obtained in the absence ((a) and (c)) or presence ((b) and (d)) of AN peroxidase. Products derived from condensation of procyanidin B1 with catechin or epicatechin were indicated as B1-C (b) and B1-EC (d), respectively.

Unlike catechin or epicatechin, there was no color change or spectrophotometric absorbance increase when using procyanidin B1 alone as a substrate. HPLC examination also indicated the lack of oxidation of procyanidin B1 alone (data not shown). The most striking feature of procyanidin B1 oxidation was that it occurred only in the presence of catechin or epicatechin. In contrast to spectra from conditions without enzyme (Figures 7(a) and 7(c)), the HPLC chromatograms clearly indicated procyanidin B1 oxidation with the formation of additional products (Figures 7(b) and 7(d)). In addition to the above-mentioned catechin homodimer, a product with an 864 Da molecular mass was formed in the presence of small amounts of catechin (Figure 7(b)). This product resulted from the condensation of procyanidin B1 with catechin with the concomitant removal of four hydrogen atoms.

In the presence of epicatechin, similar reactions occurred, that is, production of an epicatechin homodimer and an epicatechin-procyanidin B1 heterodimer (Figure 7(d)). Clearly, procyanidin B1 oxidation occurred through the condensation of one molecule of catechin or epicatechin with procyanidin B1 in place of the second molecule of catechin or epicatechin. However, a condensation reaction between two molecules of procyanidin B1 was not observed in either cases. Thus, the following reactions were indicated: catechin (or epicatechin) + procyanidin B1 + 2H_2_O_2_ → catechin-procyanidin B1 dimer (or epicatechin-procyanidin B1 dimer) + 4H_2_O.

## 4. Discussion

Most proteins in the areca nut were found in trace amounts, as determined by SDS-PAGE, with the exception of peroxidase, which was identified as the major protein component in the experiment. In addition to multiple N-glycosylation sites, a heme group was shown to exist in RPT peroxidase [10], which has a sequence similar to AN peroxidase (Figure 2(c)). The same prosthetic group, most likely resulting in the brown color with a maximum absorbance near 405 nm (Figure 1(d)), was found in AN peroxidase and therefore should be classified under the family of heme peroxidases.

AN peroxidase existed mainly as an inactive monomer at neutral pH (Figure 3(b)), while a small increase in pH, to 7.9, led to a dramatic structural change in which two identical subunits associated to form a fully active, intact protein (Figures 3(a) and 3(b)). Such observations suggested that the active site of AN peroxidase was created only when two subunits were closely associated; that is, the active site is located at the subunit interface. It was plausible that when chewing areca nut in the oral cavity, AN peroxidase is a fully active dimer in the oral extract at alkaline pH. Similar to RPT peroxidase, secretory AN peroxidase belongs to the family of class III plant peroxidases. However, AN peroxidase is structurally distinct from members of this class, which are all known as monomeric glycoproteins [9].

Peroxidase stability apparently depends on the presence of a carbohydrate moiety, as shown in studies of the recombinant protein [11, 12]. The extreme stability of AN peroxidase at room temperature was attributed to the same factor in the PNGase F digestion experiment. The glycan moiety alone had a molecular mass greater than the unglycosylated apoenzyme itself (Figure 3(c)), suggesting distribution of glycans across the surface of the whole holoenzyme molecule. The glycan moiety affected not only the enzymatic activity of the AN peroxidase but also its conformational rigidity. Without heat treatment, AN peroxidase remained intact, even after longer exposure to PNGase F (data not shown).

Peroxidase-mediated oxidation of catechins led to the formation of products with different degrees of polymerization through condensation of an A ring from one unit and a B ring from the other [13]. Two catechin dimers were reported to be the main products: colored dehydrodicatechin A and colorless dehydrodicatechin B4, with molecular masses of 576 and 578 Da, respectively [14, 15]. Comparison of the fragment ions in positive mode revealed obvious differences in the MS/MS spectra between the observed catechin dimer (see Figure S1A in supplementary Material available online at http://dx.doi.org/10.1155/2013/412851) and dehydrodicatechin A (Figure S1C).

Pourcel et al. proposed that the major oxidation product obtained with epicatechin alone was a yellow dimer named dehydrodiepicatechin A [16]. However, the MS/MS spectrum of their proposed structure (compound 6 in Figure S1E) in negative mode was significantly different from that of the epicatechin dimer in the experiment (Figure S1E). Recently, Mouls and Fulcrand reported epicatechin oxidation and demonstrated that the dimeric ions at *m/z* 579 and *m/z* 577 represented B-type and A-type dehydrodicatechin structures, respectively [17]. Prior to oxidation, epicatechin was possibly epimerized into catechin; thus, the same products were obtained, which is likely in the case of catechin oxidation. Taken together, AN peroxidase-catalyzed oxidation of catechin or epicatechin might generate dimeric structures distinct from those mentioned above, as shown by the formation of a major dimer with a different MS/MS spectrum. For the positive and negative modes, the MS/MS spectra of dimers C-C and EC-EC were analogous (Figures S1A, B, D, E), and both dimers had extremely similar structures.

In addition to catechin and epicatechin, the areca nut contained several procyanidins and their oligomeric forms [18, 19], one of which was procyanidin B1 (Figure S2A) and was identified as an AN peroxidase substrate in the experiment. The inability of procyanidin B1 alone to be oxidized by AN peroxidase is consistent with the observation that there was no procyanidin B1-B1 dimer product (Figures 7(b) and 7(d)). The active site of AN peroxidase may consist of two portions, one of which had the ability to bind to larger oligomeric substrates. In contrast, the other could only bind to monomeric units, such as catechin or epicatechin, because there was not enough space in this position to accommodate procyanidin B1 or the dimeric form of epicatechin and catechin. Notably, AN peroxidase-catalyzed oxidation was greatly inhibited in the presence of procyanidin B2, which is structurally similar to procyanidin B1; the difference is the C-3 configuration of the C ring of the epicatechin unit (Figure S2B). The extent of inhibition was proportional to the concentration of procyanidin B2 (Figures S2C, D). This observation provided evidence that C-3 on the C ring of the catechin unit in procyanidin B1 might serve as the critical reactive site for coupling with catechin or epicatechin.

There are reports indicating the presence of peroxidase in the seeds of other plants, such as soybean [20], apple, and orange [21]. Egley et al. demonstrated the possible role of peroxidase in contributing to the formation of a water-impermeable seed coat [22]. The proposed function of peroxidase is also consistent with the ability of peroxidase to form an insoluble polymer using soluble phenolics. In this study, distribution of AN peroxidase was also evaluated using zymography to examine the oral extract of the same chewer. As depicted in Figure S3 in the supplementary data, AN peroxidase was most abundant in the areca nut coat (2), at levels comparable to or even more than the amounts in the intact areca nut alone (3) or the areca nut wrapped with a Piper betle leaf coated with slaked lime (4). Notably, an additional peroxidase was detected, to a lesser extent, in the presence of Piper betle leaf (4);that is, two distinct peroxidases simultaneously acted on the oral cavity in this situation. The presence of the other peroxidase was undoubtedly the consequence of chewing Piper betle leaf coated with slaked lime (5).

In summary, further investigations remain concerning the oxidation of epicatechin and procyanidin B1, especially for the latter in the presence of catechin or epicatechin. The subject is significant to explain the inhibitory effect of procyanidin B2 on the oxidation of catechin, epicatechin, and procyanidin B1. The next goal will be to research the peroxidase in the Piper betle leaf. Due to the absence of a color change when adding hydrogen peroxide to Piper betle leaf extract, we are also searching for natural substrates in addition to the three mentioned above.

## Supplementary Material

Supplementary Figure S1. MS/MS spectra of C-C and EC-EC dimers. The dimerized ions at m/z 577 (A and B) and m/z 575 (D and E) are indicated. The MS/MS spectrum of dehydrodicatechin A in positive mode is also included (C).
Supplementary Figure S2. Inhibition of catechin and epicatechin oxidation by procyanidin B2. (A) and (B) Structures of procyanidins B1 and B2. (C) and (D) The inhibitory effect of procyanidin B2 on the oxidation of catechin (C) and epicatechin (D). The concentration of procyanidin B2 used is indicated.
Supplementary Figure S3. Zymogram detection of AN peroxidase in oral extracts. Peroxidases of the areca nut and Piper betle leaf are indicated by arrowheads. Lane 1, saliva alone as a control. Lane 2, areca nut coat. Lane 3, the intact areca nut. Lane 4, Piper betle leaf coated with slaked lime wrapped around the areca nut. Lane 5, Piper betle leaf coated with slaked lime.

## Abbreviations

AN: Areca nut
CHCA: *α*-Cyano-4-hydroxycinnamic acid
SA: Sinapinic acid
DEAE: Diethylaminoethyl
TFA: Trifluoroacetic acid.
