# Supplementary figures and images for: Peroxidase as the Major Protein Constituent in Areca Nut and Identification of Its Natural Substrates
Source: Evid Based Complement Alternat Med. 2013 Oct 24;2013:412851. doi: 10.1155/2013/412851 (PMC3821912; doi:10.1155/2013/412851)

Fig. S1


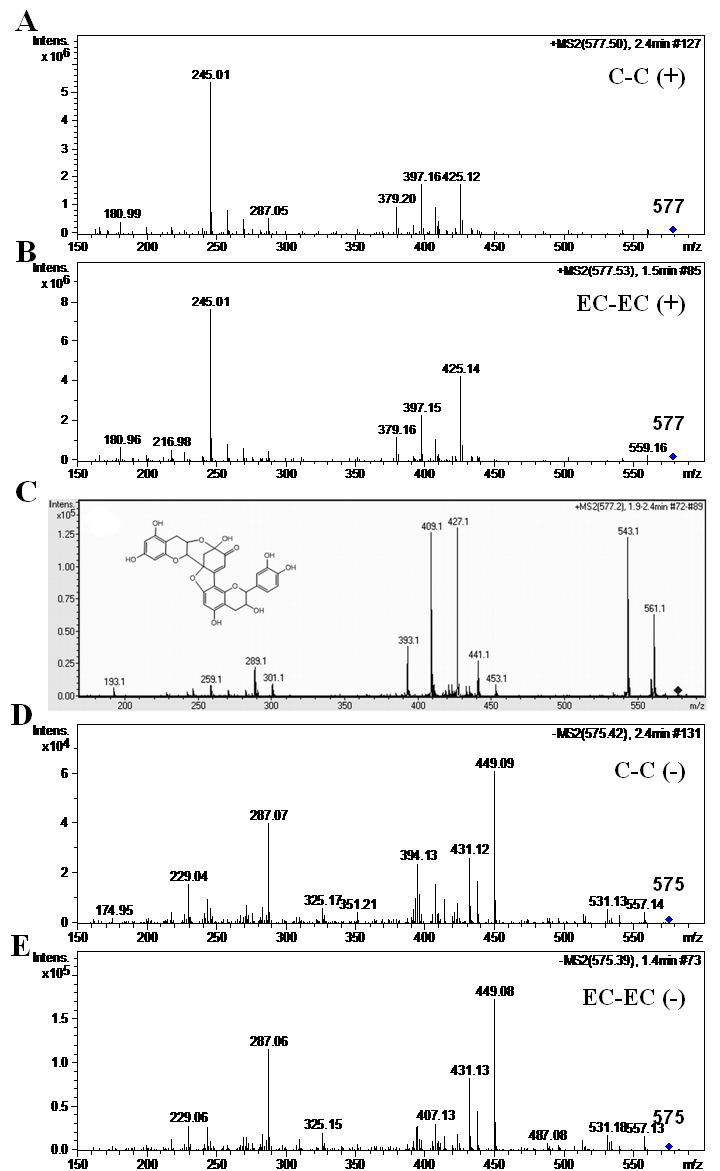


Fig. S2


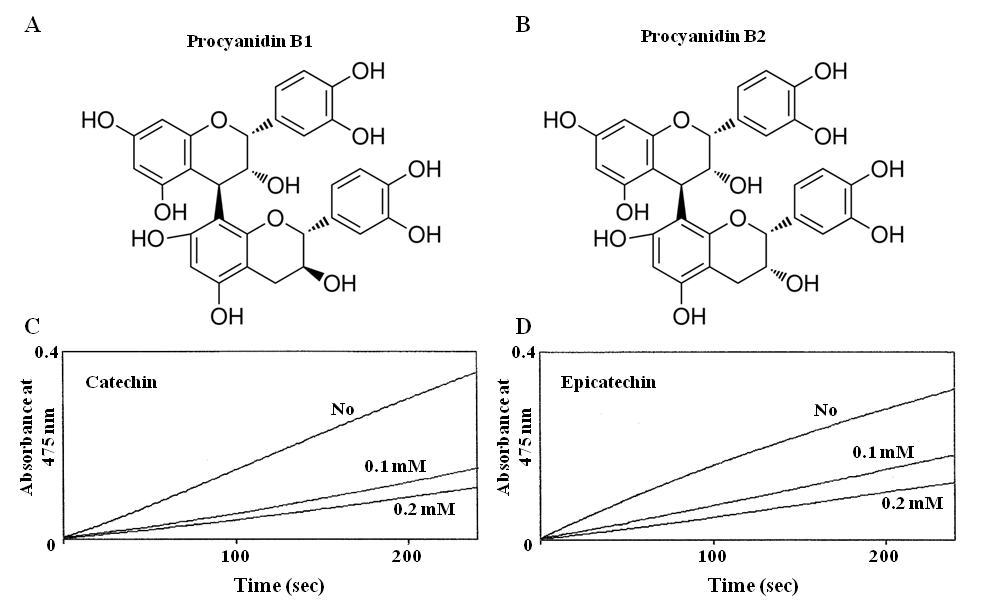


Fig. S3


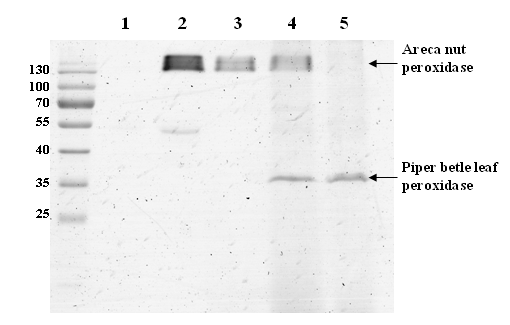

Supplement: Supplementary file 1 — Supplementary Figure S1. MS/MS spectra of C-C and EC-EC dimers. The dimerized ions at m/z 577 (A and B) and m/z 575 (D and E) are indicated. The MS/MS spectrum of dehydrodicatechin A in positive mode is also included (C). Supplementary Figure S2. Inhibition of catechin and epicatechin oxidation by procyanidin B2. (A) and (B) Structures of procyanidins B1 and B2. (C) and (D) The inhibitory effect of procyanidin B2 on the oxidation of catechin (C) and epicatechin (D). The concentration of procyanidin B2 used is indicated. Supplementary Figure S3. Zymogram detection of AN peroxidase in oral extracts. Peroxidases of the areca nut and Piper betle leaf are indicated by arrowheads. Lane 1, saliva alone as a control. Lane 2, areca nut coat. Lane 3, the intact areca nut. Lane 4, Piper betle leaf coated with slaked lime wrapped around the areca nut. Lane 5, Piper betle leaf coated with slaked lime. [file 412851.f1.doc]
